# Acquired mechanisms of immune escape in cancer following immunotherapy

**DOI:** 10.1186/s13073-018-0598-2

**Published:** 2018-11-22

**Authors:** J. Bryan Iorgulescu, David Braun, Giacomo Oliveira, Derin B. Keskin, Catherine J. Wu

**Affiliations:** 1 Department of Medical Oncology, Dana-Farber Cancer Institute, Boston, MA 02115 USA; 2 Harvard Medical School, Boston, MA 02115 USA; 3 Department of Pathology, Brigham and Women’s Hospital, Boston, MA 02115 USA; 4 Broad Institute of MIT and Harvard, Cambridge, MA 02142 USA; 5 Translational Immunogenomics Laboratory, Dana-Farber Cancer Institute, Boston, MA 02115 USA

## Abstract

Immunotherapy has revolutionized the management of numerous cancers; however, a substantial proportion that initially respond subsequently acquire means of immune escape and relapse. Analysis of recent clinical trials permits us to preliminarily understand how immunotherapies exert evolutionary pressures: selecting cancer subclones deficient in antigenicity and/or immunogenicity, thereby facilitating immune escape.

## Clinical landscape of the immune system in cancer

In recent decades, there have been exhilarating strides forward for a spectrum of advanced cancer types, many made possible by the harnessing of patients’ immune responses. Across a variety of cancers, objective responses are seen after immunotherapy in up to 50% of patients; with long-term response sustainability, in part, due to the adaptive immune system’s distinct capacity for memory. As summarized previously, multiple, largely T lymphocyte-targeted, immunotherapeutic modalities have been successfully tested in the clinic, with the most common contemporary approaches including blockade of inhibitory immune checkpoints (ICB), antigen-specific peptide vaccination, oncolytic virotherapy, and adoptive cell therapies (ACT) [1]. Substantial preclinical and clinical investigations have elucidated the favorable conditions for immunotherapy, namely: a tumor cell’s ability to properly present, or release, immunogenic antigens; an abundant neoantigen repertoire; a robust and uninhibited T-lymphocyte infiltrate; and a tumor and stromal microenvironment that permits the infiltration and functionality of effector T cells; so that activated tumor-specific T cells can identify tumor cells in the context of major histocompatibility complex (MHC)-peptide complexes and induce tumoricidal cytolysis. Cancers that employ pre-existing mechanisms to subvert any of these conditions exhibit primary resistance to immunotherapies and manifest clinically as non-responders.

**Fig. 1:**
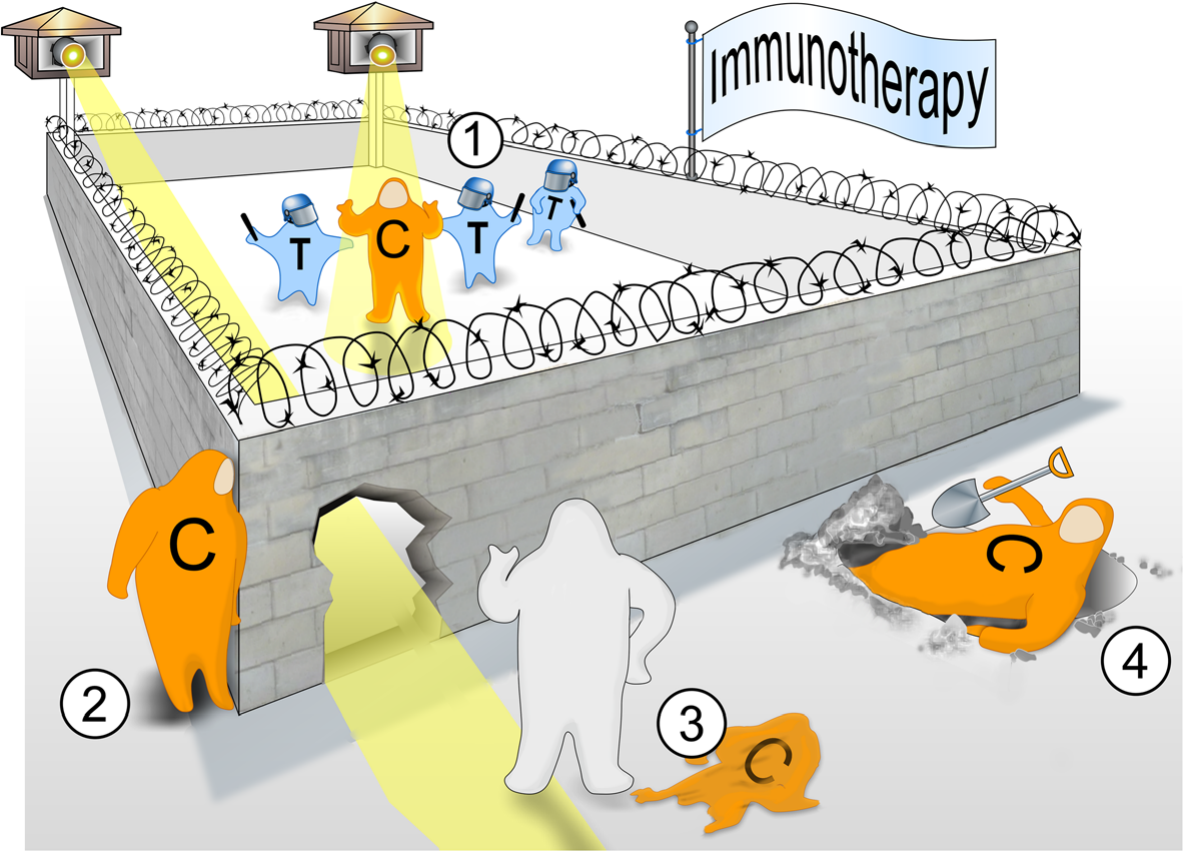
The great escape: acquired mechanisms of immune evasion in cancer. Multiple immunotherapeutic approaches have potently targeted T-cell responses (T) against cancer cells (C) in the clinical setting (1); however, a substantial subset of initial responders acquire novel immunogenomic means of immune escape and relapse. From clinical investigations, the most common acquired mechanisms of immune escape appear to be (2) deficits in antigen presentation machinery, (3) loss of antigenicity, and/or (4) loss of immunogenicity—including by exploiting bypass immune checkpoint pathways

Increasingly apparent from clinical studies across immunotherapies, however, is that at least 30–50% of cancers that initially respond subsequently acquire means of immune escape and relapse [2, 3]. Paradoxically, the patients’ cancer immunoediting mechanisms, wherein normally the adaptive immune system recognizes and eliminates immunogenic nascent tumors, may facilitate selection of cancer subclones that acquire new armaments to evade the immune responses elicited by immunotherapies. Ongoing selective pressure exerted by the immunotherapy results in immunoediting of the cancer subclones, thereby selecting for subpopulations with deficiencies in antigenicity (i.e., capacity of the antigen’s structure to specifically bind T-cell receptors (TCRs) or B-cell receptors), immunogenicity (i.e., capacity of the antigen to induce an adaptive immune response), and/or antigen presentation machinery (Fig. 1). Many of the same mechanisms of primary resistance—including deficiencies in antigenicity, immunogenicity, and antigen presentation machinery—were posited to underlie acquired resistance based on preclinical experiments; however, the rapid expansion of immunotherapy clinical trials in recent years has led to a growing clinical understanding of the diverse immunogenomic mechanisms acquired by cancers to escape patients’ immune systems and are summarized herein.

## Antigenic escape

Antigenic targets of immunotherapies, in order of increasing specificity, include cell type-specific markers of differentiation, oncofetal and cancer/testis (i.e., gamete-specific and placental-specific) antigens, and tumor-specific mutated neoantigens. Acquired loss of the cognate antigen(s) has long been associated with resistance to antigen-targeted antibody immunotherapeutics (e.g., loss of CD20 expression in B-cell lymphomas after rituximab). Similarly, tissue lineage-specific antigen-targeted approaches (e.g., Melan-A/MART-1-specific ACT or multi-melanocytic marker peptide vaccination for melanoma; and CD19-targeted chimeric antigen receptor T cell (CAR-T) or CD19-targeted bi-specific T-cell engager for B-cell acute lymphoblastic leukemia) have demonstrated the subsequent selection for and predominance of antigen-negative subclones during disease relapse [4]. Persistence of CD19-targeted CAR-T at relapse, in particular, is associated with attaining loss of that specific targeted CD19 epitope on tumor cells. In one melanoma case, diffuse T-cell infiltration following TCR-engineered ACT was associated with tumor necrosis factor (TNF)-α-mediated immunosuppressive dedifferentiation, manifested as a gain of the neural crest stem cell marker NGFR and loss of melanocytic markers [5]. Following ICB, non-small cell lung carcinoma (NSCLC) relapses have been associated with the loss of 7–18 predicted neoantigens through the elimination of subclones or deletion of chromosomal regions containing truncal alterations. These eliminated neoantigens demonstrated higher predicted binding affinities for their autologous MHC alleles and enhanced proliferative TCR responses upon stimulation of circulating lymphocytes than their retained or gained neoantigen counterparts, suggesting that these tumors were immunoediting out the most immunogenic neoantigens during immunotherapy [6]. Loss or downregulation of immunogenic neoantigens has been also seen following ICB in a melanoma case that relapsed after brief stabilization with ACT [7]. Given the potential for acquired loss of some antigens, there may be a therapeutic opportunity for complex multi-antigen vaccination-based approaches to target the immune system towards the remaining antigens that survive immunoediting. Several initial clinical trials are currently in development to investigate one such combination: ICB with multi-peptide neoantigen-specific vaccination strategies, including for breast (NCT03199040), glioblastoma (NCT02287428, NCT03422094), renal cell carcinoma (NCT02950766), melanoma, lung, and bladder cancers (NCT02897765).

## Antigen presentation machinery escape

Successful cell surface expression of the trimolecular MHC class I molecule necessitates complexing of (i) the shared structural β2-microglobulin (β2m), (ii) the α heavy chains that serve as membrane anchor and peptide binding groove (encoded by *HLA* genes), and (iii) the peptide (usually 8–10 amino acids long) within the lumen of the endoplasmic reticulum. Defects or deficiencies of any of these constituents consequently diminish MHC class I expression and antigen presentation. Since the 1990s, a small subset of cancer relapses has been noted to acquire such deficits in antigen presentation machinery following immunotherapy. The majority of these cases arose as a consequence of acquired β2m loss-of-function mutations and loss-of-heterozygosity (LOH) events, resulting in prolonged association with chaperone proteins that confine the MHC class I α chains to the endoplasmic reticulum. The predominance of acquired β2m deficits in antigen presentation machinery is likely due to the shared nature of β2m among all MHC class I molecules, whereas functional *HLA* α chain deficits require simultaneous inactivation of all co-dominantly expressed *HLA* class I alleles [2, 3, 8].

More rarely, acquired LOH events that involve the short arm of chromosome 6, which contains the *HLA-A*, *-B*, and *-C* genes, have been observed in several cancer relapses following immunotherapy, one of which demonstrated concomitant interferon (IFN)-γ-unresponsive epigenetic silencing of the remaining *HLA-A* allele by DNA methylation [9]. Notably, in 29% of relapsed acute myeloid leukemia patients who received haploidentical stem cell transplantation with infusion of donor T cells, leukemic cells lost their donor mismatched *HLA* haplotype(s), thereby evading donor T cells’ graft-versus-leukemia response [10]. Although defective peptide transport has been implicated in primary resistance to immunotherapies, there has been only one reported case of an acquired loss-of-function mutation involving peptide delivery: in the peptide-loading complex constituent tapasin (*TAPBP*) accompanied by a LOH event involving chromosome 6 [9]. Additionally, inactivating mutations with LOH of *Janus kinase 2* (*JAK2*) in one relapsed melanoma patient following ICB abrogated MHC class I and peptide transporter TAP1 upregulation in response to IFN-γ [2]. Interestingly, acquired β2m defects were also detectable in sequencing of circulating cell-free DNA in a fraction of cases, suggesting the possibility of monitoring for immune escape non-invasively [3]. As T cell-targeted immunotherapies are increasingly employed for many cancer types, the acquired loss of MHC class I expression as an immune escape route may provide an opportunity for combination immunotherapy with agents that foster natural killer (NK) cell-mediated elimination of cells lacking MHC class I expression.

## Immunogenic escape

In several cases, acquired changes in the tumor microenvironment have also been observed, including mechanisms that promoted exclusion or suppression of T cells and overexpression of extracellular matrix formation genes that prevent effective infiltration of tumors by antitumoral immune effector cells [2, 11]. In several NSCLCs that relapsed after ICB, tumors acquired upregulation of alternate immunosuppressive immune checkpoint pathways that engendered reversion to a lymphocyte-excluded state with CD8+ T cells delimited to the invasive margin of the tumor periphery [2, 11]. In two NSCLC patients treated with anti-PD-1 ICB, therapeutic antibody binding of T cells was preserved at the time of relapse, suggesting that both the persistent blocking of the PD-1 checkpoint pathway and the rise of alternative mechanisms permit immune escape [11]. At relapse, CD4+ (including FOX3P+ regulatory) and CD8+ T cells demonstrated upregulation of the TIM-3 checkpoint, particularly in those T cells that were still bound by the therapeutic PD-1 antibody. The CD8+ T cells additionally showed modest increases in expression of the CTLA-4 checkpoint. In a separate cohort of relapsed NSCLCs following anti-PD-1 with/without anti-CTLA-4, a subset also demonstrated increased expression of the immunosuppressive LAG3 and/or TIM3 checkpoints on CD3+ T cells [8]. The acquisition of alternate immune checkpoints to bypass ICB underscores the potential for combining inhibition of multiple immune checkpoint pathways to “warm” newly cold immune microenvironments.

## Conclusions

Together, clinical investigations of relapse in a spectrum of cancer types following immunotherapy have begun to identify key immunogenomic means of attaining immune escape; namely, deficits in antigen presentation machinery, loss of antigens, and exploiting alternate immune checkpoint pathways. The variety of novel acquired immune escape mechanisms highlights the potency of new immunotherapeutics to establish new, or unleash pre-existing, immune pressures, and underscores the extensive immunologic clonal diversity within cancers. Given the relatively recent availability of immunotherapies in clinical practice and the paucity of responder relapses reported in the literature, the incidence of different acquired immune escape mechanisms is difficult to estimate; however, it appears that defects in antigen presentation machinery may be more common and that loss of antigenicity may be particularly important to antigen-targeted immunotherapies. Further investigations are needed to identify and understand what the predictors, additional mechanisms, treatable targets, and roles of epigenetic regulation are in acquired immune resistance. These constraints highlight the critical need for incorporating longitudinal and postmortem sampling into clinical trial designs for immunotherapies—particularly at the time of disease relapse or progression—in order to better understand the cancers’ primary and adaptive resistance mechanisms and whether there are new (and targetable) acquired mechanisms of immunoresistance. Novel immunogenomic tools (e.g., single-cell RNA sequencing, mass cytometry, multiplexed ion beam imaging, etc.) allow for an unprecedented, detailed dissection of the tumor-immune microenvironment at the time of acquired resistance. A substantial proportion of cancer patients that initially respond to immunotherapy will acquire novel mechanisms of immune escape that result in tumor relapse. Understanding the immunogenomic mechanisms of acquired resistance will be vital for identifying opportunities to rationally combine different modalities and scheduling of immunotherapies, and for expanding the successes of novel immunotherapies to more cancer patients.

## Abbreviations

ACT: Adoptive cell therapies
ICB: Immune checkpoint blockade
LOH: Loss-of-heterozygosity
MHC: Major histocompatibility complex
NSCLC: Non-small cell lung carcinoma
TCR: T-cell receptor
β2m: β2-microglobulin
